# 548. A Randomized, Double-blind, Controlled Trial of Vancomycin Taper/Pulse and Fidaxomicin compared to Vancomycin for Treatment of a First or Second Recurrence of Clostridioides difficile Infection

**DOI:** 10.1093/ofid/ofaf695.021

**Published:** 2026-01-11

**Authors:** Stuart Johnson, Dale N Gerding, Curtis Donskey, Hua Feng, Neil Johnson, Michelle Johnson, Kimberly M Carlson, Ling Ge, Alexa M Goldberg, Domenic J Reda, Michael W Climo, Kalpana Gupta, Matthew B Goetz

**Affiliations:** Hines VA Hospital and Loyola University Medical Center, Hines, Illinois; Edward Hines Jr. VA Hospital, Hines, Illinois; Cleveland VA Hospital, Cleveland, Ohio; Edward Hines Jr. VA Hospital, Hines, Illinois; Edward Hines Jr. VA Hospital, Hines, Illinois; Edward Hines Jr. VA Hospital, Hines, Illinois; Edward Hines Jr. VA Hospital, Hines, Illinois; Edward Hines VA Hospital, Hines, Illinois; Albuquerque VA Medical Center, Albuquerque, New Mexico; University of Illinois at Chicago, Chicago, Illinois; Richmond VA Medical Center, Richmond, Virginia; VA Boston Healthcare System and Boston Universiy School of Medicine, West Roxbury, MA; VA Greater Los Angeles Healthcare System, Los Angeles, California

## Abstract

**Background:**

Vancomycin taper followed by pulse and fidaxomicin are two common treatments for recurrent *C. difficile* infection (rCDI) but they have not been formally compared.

**Figure:**
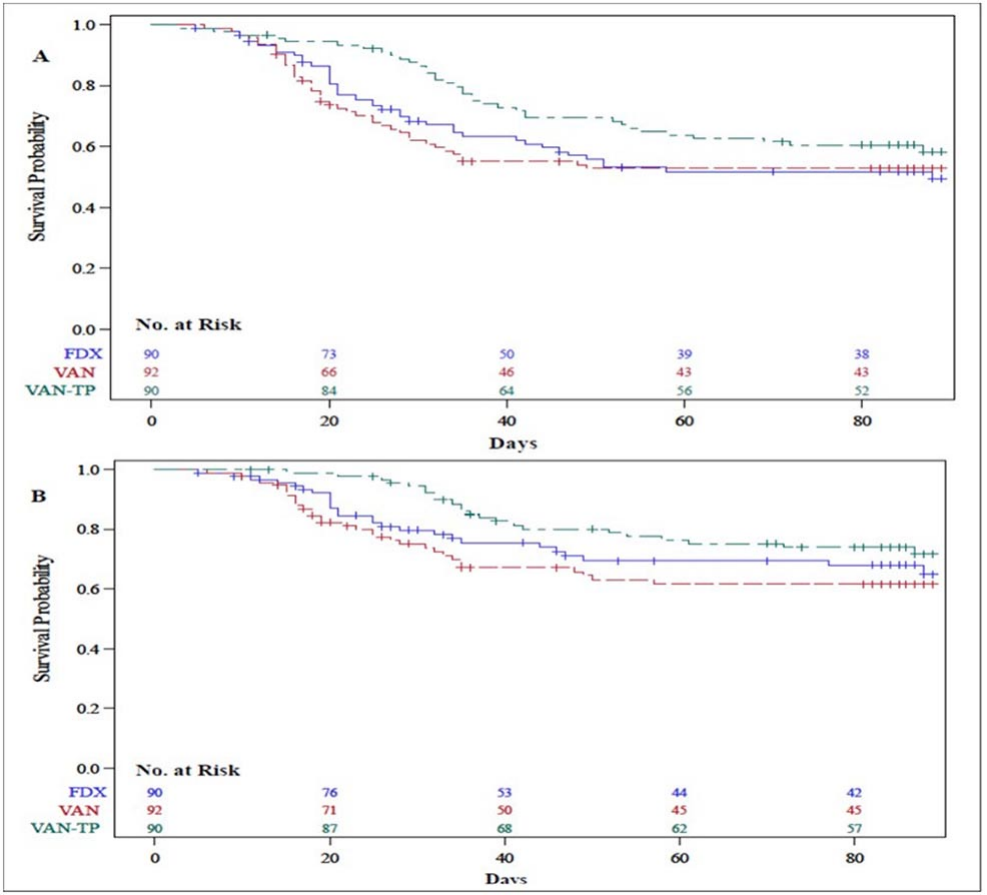
Kaplan-Meier plot of days from symptom resolution to recurrences of diarrhea [A] and recurrent C. difficile infection (rCDI) [B] in the modified intent-to-treat population.

**Methods:**

We conducted a randomized, double-blind trial of vancomycin taper and pulse (VAN-TP, 125 mg four times daily for 10d, followed by a once daily, once every other day, and once every third day, each for 7 days) and fidaxomicin (FDX, 200 mg twice daily for 10d) compared to vancomycin (VAN, 125 mg four times daily for 10d) for patients with a first or second rCDI episode. The primary study endpoint was sustained clinical response (SCR) at day 59 using a diarrhea composite outcome (D-COM) in modified intent to treat analysis (mITT). Secondary outcomes included SCR without rCDI (CDI-COM), diarrhea resolution at day 10, recurrent diarrhea and rCDI.

**Results:**

308 participants were randomized to 1 of the 3 arms. Sustained D-COM was higher for VAN-TP (58.6%) than for VAN (44.1%) at day 59 (Unadjusted P=0.04, Z-statistic scores at 2.03-2.08, efficacy boundary 2.234 controlling the FWER at 0.05, mITT). Sustained CDI-COM was also higher for VAN-TP (64.8%) than for VAN (48.4%) (P=0.05). There was no difference in D-COM or CDI-COM at day 59 between FDX and VAN (D-COM: 44.1% vs. 44.1%, P=1.00; CDI-COM: 51.8% vs. 48.4%, P=0.86). Symptom resolution by day 10 was greater than 90% for all three groups. CDI recurrence, but not diarrhea recurrence was less common in VAN-TP than in VAN at day 59 (rCDI: 26.6% versus 43.6%, P=0.05; diarrhea recurrence: 34.1% vs. 49.4%, P=0.08). Survival estimates for days from symptom resolution to diarrhea recurrence (Fig A) and CDI recurrence (Fig B) show separation by day 20 which continues to day 90 for VAN-TP compared to VAN.

**Conclusion:**

A tapered and pulsed regimen of vancomycin following a standard treatment course was superior to a standard course of vancomycin for sustained resolution of diarrhea and CDI at day 59 in patients with recurrent CDI. A standard course of fidaxomicin was not different from a standard course of vancomycin in this population.

**Disclosures:**

All Authors: No reported disclosures

